# Structural Damage Classification in a Jacket-Type Wind-Turbine Foundation Using Principal Component Analysis and Extreme Gradient Boosting

**DOI:** 10.3390/s21082748

**Published:** 2021-04-13

**Authors:** Jersson X. Leon-Medina, Maribel Anaya, Núria Parés, Diego A. Tibaduiza, Francesc Pozo

**Affiliations:** 1 Control, Modeling, Identification and Applications (CoDAlab), Department of Mathematics, Escola d’Enginyeria de Barcelona Est (EEBE), Campus Diagonal-Besòs (CDB), Universitat Politècnica de Catalunya (UPC), Eduard Maristany 16, 08019 Barcelona, Spain; jersson.xavier.leon@upc.edu; 2 Departamento de Ingeniería Mecánica y Mecatrónica, Universidad Nacional de Colombia, Cra 45 No. 26-85, Bogotá 111321, Colombia; 3 MEM (Modelling-Electronics and Monitoring Research Group), Faculty of Electronics Engineering, Universidad Santo Tomás, Bogotá 110231, Colombia; maribelanaya@usantotomas.edu.co; 4 Laboratori de Càlcul Numèric (LaCàN), Department of Mathematics, Escola d’Enginyeria de Barcelona Est (EEBE), Campus Diagonal-Besòs (CDB), Universitat Politècnica de Catalunya (UPC), Eduard Maristany 16, 08019 Barcelona, Spain; 5 Departamento de Ingeniería Eléctrica y Electrónica, Universidad Nacional de Colombia, Cra 45 No. 26-85, Bogotá 111321, Colombia; dtibaduizab@unal.edu.co; 6 Institute of Mathematics (IMTech), Universitat Politècnica de Catalunya (UPC), Pau Gargallo 14, 08028 Barcelona, Spain

**Keywords:** structural health monitoring, principal component analysis, extreme gradient boosting, machine learning, classification, wind-turbine foundation

## Abstract

Damage classification is an important topic in the development of structural health monitoring systems. When applied to wind-turbine foundations, it provides information about the state of the structure, helps in maintenance, and prevents catastrophic failures. A data-driven pattern-recognition methodology for structural damage classification was developed in this study. The proposed methodology involves several stages: (1) data acquisition, (2) data arrangement, (3) data normalization through the mean-centered unitary group-scaling method, (4) linear feature extraction, (5) classification using the extreme gradient boosting machine learning classifier, and (6) validation applying a 5-fold cross-validation technique. The linear feature extraction capabilities of principal component analysis are employed; the original data of 58,008 features is reduced to only 21 features. The methodology is validated with an experimental test performed in a small-scale wind-turbine foundation structure that simulates the perturbation effects caused by wind and marine waves by applying an unknown white noise signal excitation to the structure. A vibration-response methodology is selected for collecting accelerometer data from both the healthy structure and the structure subjected to four different damage scenarios. The datasets are satisfactorily classified, with performance measures over 99.9% after using the proposed damage classification methodology.

## 1. Introduction

Structural health monitoring (SHM) allows identifying the states of structures to prevent damage that can occur because of operational and/or environmental conditions. It is possible to detect the beginning of a possible damage/failure in a structure and its components using methods associated with SHM systems [1]. Damage identification includes several levels including damage detection [2,3]. However, disturbances for robust damage identification need to be considered using algorithms for data-driven strategies [4]. Damage detection under changing environmental and operational conditions (EOC)—as in reality—is very complicated because the damage effects on the measured signals are masked by the EOC effects on the signals. The environmental conditions to which the structure is subjected has a stochastic nature; hence, the aim is to develop reliable methods for monitoring structures [5].

Wind power plants are a good example of systems exposed to environmental influences. Offshore wind power is clean energy that exploits high and uniform wind speed conditions with even larger offshore wind turbines [6]. However, it is necessary to lower the operations and maintenance (O&M) costs by anticipating potential wind-turbine damage. Therefore, SHM systems are a helpful tool in the wind power industry that help provide early damage alerts and reduce maintenance costs [7].

Recently, sensors and signal processing techniques have been successfully applied for the analysis and evaluation of structures for obtaining significant and reliable results when the state of structures is evaluated [8]. The EOC effects on signals can be filtered or considered using pattern-recognition techniques. These data-based methods rely on pattern recognition and artificial intelligence techniques to differentiate a healthy structure from a damaged one.

Damage diagnosis for offshore wind-turbine foundations remains an open field of research. Common SHM approaches are based on guided waves with a known input excitation; however, in this type of structure, the applicability of guided waves is not functional because external perturbation effects caused by wind and marine waves are ignored. An approach based only on vibration-response accelerometer signals needs to be considered to address the challenge of online and in-service SHM for wind turbines. Some variants of the vibration-response-only SHM strategy for wind-turbine foundations have been reported. Vidal et al. [7] developed a data-driven approach with the following four stages: the wind is simulated as Gaussian white noise [9] and the data from accelerometers are collected; the data are pre-processed via group-reshape and column-scaling; a feature extraction approach based on principal component analysis (PCA) is used; and finally, *k* nearest neighbors (*k*NN) and quadratic-kernel support vector machine (SVM) [10] are tested as classifiers. The best classification accuracy is obtained using the SVM algorithm, and it reaches 99.9%.

In contrast to the conventional data-driven SHM techniques, the deep learning approach has been demonstrated its successfully application to solve SHM problems [11,12]. An approach based on a deep learning strategy via convolutional neural networks (CNNs) was presented in [13]. The deep learning approach is based on the signal-to-image conversion of the accelerometer data to gray-scale multichannel images with as many channels as the number of sensors in the condition monitoring system. Furthermore, it is based on a data augmentation strategy to diminish the test set error of the deep CNN used to classify the images. The CNN comprises seven convolutional layers performing feature extraction, followed by three fully connected layers and a SoftMax block for classification; an overall accuracy of 99.9% is obtained. Hoxha et al. [14] solved the identification and classification damage problem in an experimental laboratory wind-turbine offshore jacket-type foundation through a fractal dimension methodology that performs feature extraction in a machine learning (ML) setting. *k*NN, quadratic SVM, and Gaussian SVM were used as classifiers. The best algorithm was found to be the Gaussian SVM, which achieved a classification accuracy of 98.7%.

This research seeks to solve the damage classification problem of in situ real structures exposed to strong changes in EOCs (e.g., offshore wind power plants) using ML algorithms that use signals from sensor networks as inputs. The main goal of data-driven algorithms is to analyze large or complex sensor networks that provide multivariate information using ML approaches. These complex sensor networks can be found in some SHM solutions [15,16], classification of gases by means of electronic noses [17,18], and classification of liquids by means of electronic tongues [19], among others. A common problem for data-driven algorithms is that data captured by the network of sensors have a high dimensionality [20], and therefore, algorithms are employed to handle and process this large amount of information. Within the pattern-recognition process, the extraction of both linear and nonlinear characteristics reduces the dimensionality of the original data by eliminating redundant characteristics and noise from the sensor signals [21]. Linear and nonlinear manifold learning algorithms [22] can be used in the feature extraction stage and as subspace learning algorithms [23] to minimize intraclass distances and maximize distances between classes in a clustering or cluster problem. This facilitates the classification of the ML algorithm, which can be unsupervised, semi-supervised, or supervised.

Different strategies have been used to solve structural damage-detection problems. There are traditional, machine learning methods with their parametric and non-parametric variants and deep learning methods [24]. In 2020, Gardner et al. presents the power of machine learning with methods such as compressive sensing and transfer learning to solve different structural analysis [25]. In Chandrasekhar et al., a machine learning approach is used to solve SHM in operational wind-turbine blades. This work uses Gaussian processes (GPs) to predict the edge frequencies of one blade given that of another to identify the healthy state of the blade [26]. A systematic review of machine learning algorithms in structural health monitoring is presented in [27]. That work highlights the importance of data manipulation in machine learning tasks, including topics as data cleaning and feature engineering.

Previous studies focused on structural damage classification using multisensor systems; they used several ML algorithms and data reduction for pattern recognition. For instance, PCA [28], self-organizing maps (SOM) [29], *k*NN [30], artificial immune systems (AIS) [31], SVMs [32], and *t*-distributed stochastic neighbor embedding (t-SNE) [33,34]. This study presents a structural damage classification methodology for pattern recognition and signal processing in sensor networks that achieves good classification performance and advantages in calculation time to continue the improvement and development of damage classification methodologies. This methodology is composed of different stages: normalization of the signal considering the differences in magnitudes obtained by the sensors; linear feature extraction; and dimensionality reduction via the PCA method to form a feature vector that will serve as input to an extreme gradient boosting ML classifier algorithm. This methodology was validated using a small-scale wind-turbine jacket foundation. In addition, a 5-fold cross-validation procedure was performed to determine the average classification performance measures of an unbalanced classification problem showing excellent results.

The remainder of this paper is organized as follows. Section 2 describes the main problem considered in the current work. Then, in Section 3 and Section 4, the proposed SHM strategy is described in detail. Section 3 lists the steps performed in the training data preparation. Section 3 describes the small-scale wind-turbine foundation with all its parts, the excitation system, sensors, and data acquisition process. Then, Section 4 describes the XGBoost classifier as an ensemble method (Section 4.1). Section 5 summarizes the flow of the real-time classification of a new observation from a wind turbine (WT) that must be diagnosed. In Section 6, the obtained results are compiled, and they indicate exceptional performance for all considered metrics. Finally, the main conclusions are presented in Section 7, in addition to the future research directions.

## 2. Problem Statement

**Figure 1:**
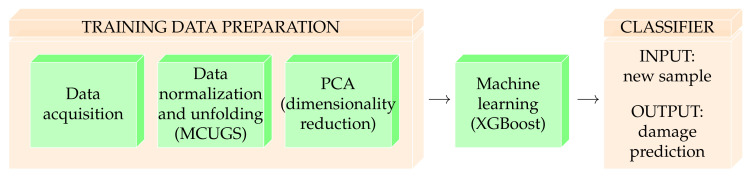
Structural damage classifier construction process.

This study aims to provide an accurate structural damage classification methodology that can specify whether a wind-turbine foundation is damaged; if it is damaged, the methodology can detect the nature of the damage. The classification methodology is derived by first obtaining a training set of data from both damaged and non-damaged structures (data acquisition). These data are properly pre-processed (data normalization and unfolding), and data transformation and dimensionality reduction techniques are applied to discard features that are not relevant to the classification problem (linear feature extraction). These new small-dimensional data are used as inputs to train the supervised machine learning classifier. Once the classification methodology is employed, given a new experimental sample of the wind-turbine foundation, the classification algorithm can predict the structural state of the foundation. Figure 1 illustrates the process used to obtain the damage classifier.

Before using the classification algorithm to classify new data, it is crucial to evaluate the performance of the algorithm. This evaluation is a challenging problem because the available data samples must be used to define the classifier and estimate its performance when making predictions of new samples. The training dataset and test set must be sufficiently large and representative of the underlying problem so that the resulting performance of the classifier is not too optimistic or pessimistic. In fact, if the collected dataset is very large and representative, one can split the dataset into two parts and use the first part to train the model and the second part to test it. However, this is rarely the case, and it is standard to use a *k*-fold cross-validation error estimation method.

**Figure 2:**
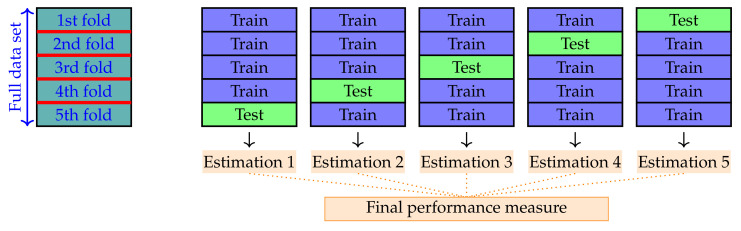
Model validation using 5-fold cross-validation.

The basic idea of this procedure is to split the full dataset into *k* folds (or subsets). This allows the generation of *k* models, each of which takes the data from k−1 folds to train the algorithm and use the remaining data to set its performance. The overall performance of the model is calculated from the mean of the estimates of these runs (see Figure 2).

## 3. Data Preprocessing: Training Data Preparation

This section details and provides a theoretical background for each step of the training data preparation developed in this work, as shown in Figure 1. The experimental setup is described succinctly in Section 3.1.

### 3.1. Experimental Setup

**Figure 3:**
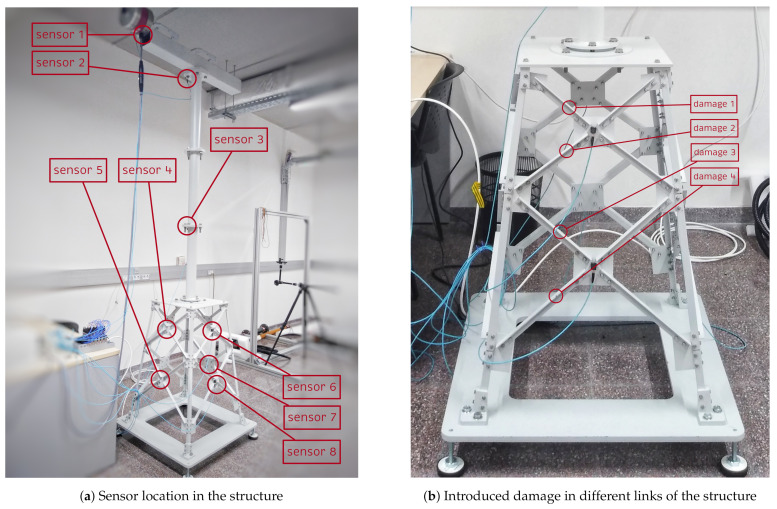
Small-scale wind-turbine foundation.

The structure of a small-scale wind-turbine foundation is used to validate the damage detection and localization methodology developed in this study. This benchmark structure, based on the one designed by Zugasti [35], was considered by Hoxha et al. [36], Vidal et al. [7], and Puruncajas et al. [13] to validate different approaches for structural damage detection and classification experimentally. The benchmark structure—placed in the CoDAlab laboratory (Escola d’Enginyeria de Barcelona Est, Universitat Politècnica de Catalunya, Barcelona, Spain)—is shown in Figure 3a; it is composed of three parts: (i) the nacelle on the top, (ii) tower in the middle, and (iii) jacket on the bottom. The total height of the structure is 2.7 m. The jacket was made of steel bars and bolts. A 5 mm crack was introduced at four different bars located in the jacket structure, one at a time. It can be said that the 5 mm crack damage is small for this structure [35] and therefore hard to detect. Summarizing, four different damage scenarios are considered in this study, as shown in Figure 3b.

Data used to validate the structural damage classification methodology were obtained using the following experimental process. First, an arbitrary function generator (GW INSTEK AFG-2005) was used to apply a white noise signal to the structure. Four different wind speeds were simulated by applying different amplitudes of the white noise signal with factors of 0.5,1,2, and 3. Then, the white noise signal was amplified and applied to an inertial shaker (GW-IV47 from Data Physics) located at the nacelle top beam to simulate the external perturbation effects caused by wind and marine waves. This signal produces a vibration response that is captured using eight triaxial accelerometers (PCB Piezotronics, Model 356A17) with a sensitivity: (±10%) 500 mV/g (51 mV/(m/s${}^{2}$)). The location of the eight sensors was determined using the Sensor Elimination by Modal Assurance Criterion (SEAMAC) method [35], and their final location is shown in Figure 3a. Taking as reference Figure 3b as the front view of the structure, below in Table 1 the location of each of the 8 sensors is detailed. Finally, the data acquisition process was performed using a cDAQ-9188 chassis from National Instruments and six NI-9234 modules, each of which has four channels, thereby conforming to a total of 24 channel data acquisition systems per time instant.

### 3.2. Data Acquisition

Data were obtained by performing several experiments applying different white noise signals to the small-scale wind-turbine foundation, both to the undamaged structure and the four different damaged structures. Table 2 show the five labels associated with each structural state, the number of tests per structure for a particular amplitude of the input signal, and the total number of tests related to the four excitation amplitudes that simulate the behavior of wind and marine waves (0.5,1,2, and 3, respectively). The total number of experiments was 5740, divided into five classes. The data are slightly imbalanced because the number of samples per class is not equal (2460 undamaged data samples versus 820 samples per damage class). Therefore, special care should be ensured when computing the performance measures of the classification algorithm.

Each test had a duration of 8.789 s using a sampling frequency of 275 Hz to acquire the acceleration measures; 2417 time instant measures were obtained for each of the 24 sensors.

**Figure 4:**
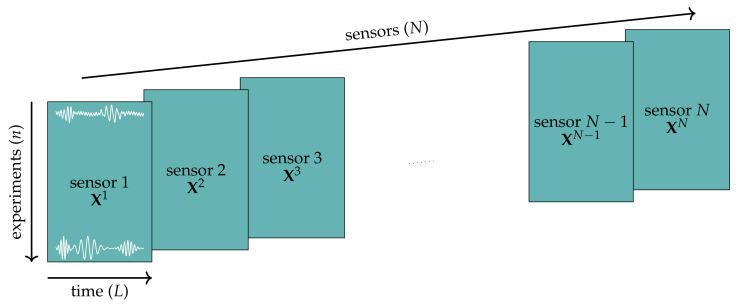
Initial arrangement of the collected data for mean centering unitary group scaling.

These collected data are arranged into a three-dimensional matrix of size n×L×N, where n=5740 stands for the total number of tests, L=2417 stands for the number of time measures, and N=24 stands for the number of sensors (8 triaxial accelerometers), as shown in Figure 4. For normalization purposes, sensor measures are grouped into n×L matrices $X^{1},X^{2},\ldots ,X^{N}$.

### 3.3. Data Normalization: Mean-Centered Unitary Group Scaling

A prerequisite for most linear and nonlinear extraction techniques is obtaining *mean-centered* data. This requires the columns of matrix $X^{k},\;k=1,\ldots ,N$ to have zero mean for each sensor. Because the data acquired in the wind-turbine foundation come from several sensors, they can have different magnitudes; therefore, scaling the data is key before analyzing it. Among all existing normalization techniques, the mean-centered group-scaling method (MCGS) [19,37,38,39] demonstrated very accurate behavior.

The MCGS group-scaling technique is devised as a two-step procedure. First, the columns of all matrices are modified by computing the mean of each column and subtracting this mean from its corresponding column values. This procedure is also called column-wise scaling [7,36]. For each sensor k=1,…,N and time instant j=1,…,L, we define

(1) $$\begin{array}{c} \mu_{j}^{k}=\mathrm{mean}\left({\mathrm{col}}_{j}\left(X^{k}\right)\right)=\frac{1}{n}\sum_{i=1}^{n}x_{ij}^{k}, \end{array}$$

to be the mean of all measures acquired by sensor *k* at time instant *j*; $x_{ij}^{k}$ is the (i,j)th element entry of matrix $X^{k}$, ${\mathrm{col}}_{j}\left(X^{k}\right)$ is the *j*th column of matrix $X^{k}$, and it introduces the mean-centered matrices ${\overset{˘}{X}}^{k}$ defined as

$${\mathrm{col}}_{j}\left({\overset{˘}{X}}^{k}\right)={\mathrm{col}}_{j}\left(X^{k}\right)-\mu_{j}^{k}\;1_{n},$$

where $1_{n}$ denotes the all-ones *n*-dimensional column vector.

Then, the matrix ${\overset{˘}{X}}^{k}$ is scaled by dividing all data by the standard deviation of all sensor *k* measurements. Thus, the elements of ${\overset{˘}{X}}^{k}$ are divided by

$$\sigma_{\mathrm{MCGS}}^{k}=\mathrm{std}\left(X^{k}\right)=\sqrt{\frac{1}{nL}\sum_{i=1}^{n}\sum_{j=1}^{L}{\left(x_{ij}^{k}-\mu^{k}\right)}^{2}},$$

where $\mu^{k}$ denotes the mean of all sensor *k* measurements, i.e., $\mu^{k}=\left(\sum_{j=1}^{L}\mu_{j}^{k}\right)/L$.

In this study, a novel alternative to the MCGS scaling technique is considered to yield normalized data with both sensor-block and global unitary standard deviation. The key idea is to scale the mean-centered data using the standard deviation, i.e.,

(2) $$\begin{array}{c} \sigma_{\mathrm{MCUGS}}^{k}=\mathrm{RMS}\left({\overset{˘}{X}}^{k}\right)=\sqrt{\frac{1}{nL}\sum_{i=1}^{n}\sum_{j=1}^{L}{\left({\overset{˘}{x}}_{ij}^{k}\right)}^{2}}=\sqrt{\frac{1}{nL}\sum_{i=1}^{n}\sum_{j=1}^{L}{\left(x_{ij}^{k}-\mu_{j}^{k}\right)}^{2}}, \end{array}$$

where the standard notation for the (i,j)th element entry of matrix $\overset{˘}{X^{k}}$, ${\overset{˘}{x}}_{ij}^{k}$, is used. The difference between MCGS and the new mean-centered unitary group-scaling technique is the definition of the block standard deviation $\sigma^{k}$: the MCGS method subtracts the global block mean value of the data, whereas the MCUGS subtracts the mean value of the corresponding column. As mentioned before, the standard deviation of both the total MCUGS normalized data and the data contained in each sensor block is 1. In engineering applications where the mean values per column $\mu_{j}^{k}$ are nearly constant in a block and therefore close to $\mu^{j}$, MCGS and MCUGS produce nearly equal normalized data. However, in applications where the mean values per column have large variations, the final standard deviation of the MCGS method may not be close to 1.

For simplicity, the MCUGS matrices ${\overset{˘}{X}}^{k}$ are renamed simply as $X^{k}$; in other words, the matrix values $x_{ij}^{k}$ are redefined without changing the notation as $x_{ij}^{k}:=\left(x_{ij}^{k}-\mu_{j}^{k}\right)/\sigma_{\mathrm{MCUGS}}^{k}$.

### 3.4. Data Unfolding

After normalizing the data, the three-dimensional matrix *experiments* × *time instants* × *sensors* is rearranged into a two-dimensional matrix *experiments* × (*time instants* × *sensors*). The sensor matrices $X^{k}$ are placed one after another so that the data are collected into a single n×(L·N) matrix.

(3) $$\begin{array}{c} X=(X^{1},X^{2},\ldots ,X^{N}). \end{array}$$

Each row of matrix X is an L·N=2470× 24=58,008 vector that contains the data associated with a particular test (2470 times instances per 24 sensors). The *i*th row of matrix X that collects the information of all sensors for the *i*th test is

(4) $$\begin{array}{c} {\mathrm{row}}_{i}\left(X\right)=\left(x_{i1}^{1},x_{i2}^{1},\ldots ,x_{iL}^{1},x_{i1}^{2},x_{i2}^{2},\ldots ,x_{iL}^{2},\ldots ,x_{i1}^{N},x_{i2}^{N},\ldots ,x_{iL}^{N}\right),\;i=1,\ldots ,n=5740. \end{array}$$

**Figure 5:**
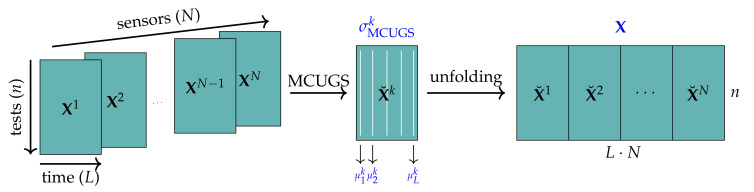
Initial data arrangement, MCUGS, and unfolding.

These rows (5740 rows describing 58,008 features) are the input samples for the feature extraction technique described in Section 3.5 for obtaining transformed samples with a low number of features that will be the input of the data-driven classification methodology. The process of data arrangement, normalization, and unfolding is illustrated in Figure 5.

### 3.5. Data Transformation and Data Reduction: Feature Extraction

Let $X\in R^{n\times D}$, where D=L·N denotes the total number of features of the data and is the matrix that collects all samples defined in Equation (3). Feature extraction techniques aim to discover a low-order model of dimension d≪D, which properly represents the variability of X, thereby eliminating redundancies. The aim is to determine a reduced *d*-dimensional manifold described by the transformed matrix $Y\in R^{n\times d}$ retaining most of the information or variance of the original matrix X, where the variance of a matrix (which coincides with its squared Frobenius norm) is defined as

$$\begin{array}{c} \mathrm{var}\left(X\right)=\mathrm{trace}\left(X^{⊤}X\right)={∥X∥}_{F}^{2}. \end{array}$$

The PCA is one of the most effective techniques for-dimensionality reduction. This technique discovers linear manifolds that characterize the data by diagonalizing the covariance matrix $C=\frac{1}{n-1}X^{⊤}X=U\Lambda U^{⊤}$, where the diagonal terms of Λ are $\lambda_{1}>\lambda_{2}>\cdots >\lambda_{D}$, and retaining only *d* eigenvectors of the decomposition yields

$$Y=X\;{\mathrm{col}}_{1..d}\left(U\right),$$

where the matrix ${\mathrm{col}}_{1..d}\left(U\right)$ contains only the first *d* columns of matrix U associated with the *d* eigenvectors with the largest eigenvalues. Because the total variance is given by the sum of all eigenvalues multiplied by n−1,

$$\begin{array}{cc} \mathrm{var}(X) & =\left(n-1\right)\sum_{i=1}^{D}\lambda_{i}, \end{array}$$

and since

$$\begin{array}{cc} \mathrm{var}(Y) & =\left(n-1\right)\sum_{i=1}^{d}\lambda_{i}, \end{array}$$

it is straightforward to compute the value of the variance retained by the PCA method as

$$\begin{array}{c} \frac{\sum_{i=1}^{d}\lambda_{i}}{\sum_{i=1}^{D}\lambda_{i}}. \end{array}$$

In general, linear and nonlinear manifold learning algorithms are based on the idea that the high dimensionality of the dataset is artificial, and that the samples are characterized by a low-dimensional manifold (or subspace) embedded in a high-dimensional space [40]. Manifold learning algorithms are used to identify these meaningful low-dimensional structures in the data, which provides a small number of relevant features that serve as input for the machine learning classification algorithms.

Manifold learning techniques introduce a transformation or map *f* from $R^{D}$ to $R^{d}$ such that the new samples are defined by $y_{i}=f\left(x_{i}\right)$. To this end, samples associated with the original and low-dimensional model are denoted as $x_{i}^{⊤}={\mathrm{row}}_{i}\left(X\right)\in R^{D}$—where ${\mathrm{row}}_{i}\left(X\right)$ stands for the *i*th row of matrix X— and $y_{i}^{⊤}={\mathrm{row}}_{i}\left(Y\right)\in R^{d}$ for i=1,…,n, respectively. In the current application, the PCA method is recovered by setting *f* to be the linear transformation given by $y_{i}=f\left(x_{i}\right)=x_{i}^{⊤}{\mathrm{col}}_{1..d}\left(U\right)$.

The *d* components of the vector $y_{i}$ are the features $F_{j},\;j=1,\ldots ,d$, which are inputs of the classifier described in Section 4. The *d* features associated with the *i*th sample are

(5) $$\begin{array}{c} F_{j}^{i}=y_{i}^{⊤}e_{j}, \end{array}$$

where $e_{j}\in R^{d}$ is the *j*th vector of the canonical basis.

## 4. Damage Detection and Classification Procedure: Extreme Gradient Boosting

This section explains the main characteristics of the XGBoost classifier. First, a detailed explanation of the XGBoost classifier as an ensemble method (providing a forest of regression trees) is provided, followed by a description of the main XGBoost parameters (see Section 4.2). Finally, Section 4.3 presents the validation procedure for evaluating the performance of the classifier.

### 4.1. XGBoost as an Ensemble Method

Currently, one of the most popular and accurate machine learning classifiers is the extreme gradient boosting technique (XGBoost) [41]. The gradient boosting method [42] can improve speed and performance. This section briefly describes the main characteristics of the XGBoost method. Readers are referred to [41,43] for fully detailed explanations.

The XGBoost method is an ensemble method that involves sequentially creating and adding regression trees. New trees are created to correct the errors in the predictions from the existing set of trees, which boosts the attributes that lead to the misclassification from the previous tree. Thus, multiple trees are built on top of each other to correct the errors in the previous tree. The XGBoost classifier thus provides a *forest* of regression trees, where the prediction of the forest is a weighted average of the predictions of its trees.

XGBoost exploits the limit of computational resources in the gradient boosting decision tree algorithm [42]. The boosting approach employs random sampling to train several classifiers, and then, the classifiers are assembled to synthesize a higher-performance classifier [44]. Boosting assigns a weight to each observation and modifies the weight after training a classifier. Observations with modified weights are employed to train the next classifier. In addition, the gradient boosting method focuses on the gradient reduction of the loss function in the previous tree-trained models. XGBoost is a scalable tree boosting system that exploits a weighted quantile sketch for approximate tree learning [41].

Some important characteristics that make the XGBoost classifier one of the best classifier algorithms are the use of inbuilt cross-validation, which handles missing values; the use of regularization to avoid overfitting in the model; a novel tree learning algorithm for handling sparse data; save resources and time with a cache access pattern mechanism; and incorporate sharding and data compression.

**Figure 6:**
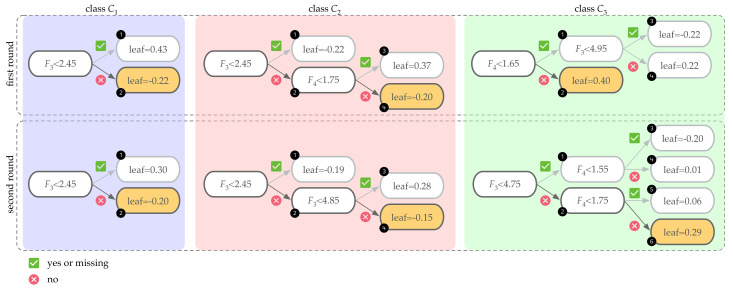
Forest of R·l=2·3=6 trees, where R=2 is the number of boosting rounds and l=3 is the number of classes. Given a specific sample with features $F_{1}=10.24,F_{2}=3.57,F_{3}=5.02$, and $F_{4}=2.38$, the decision rules of the forest predict that this sample is associated with the leaves {2,4,2} of the first round and to the leaves {2,4,6} in the second round.

The use of the XGBoost technique for multiclass classification purposes is shown here for a particular example of a sample composed of 3 classes, $\left\{C_{1},C_{2},C_{3}\right\}$; four features $\left\{F_{1},F_{2},F_{3},F_{4}\right\}$ (continuous real values); and a forest obtained using an ensemble of two boosting rounds (see Figure 6). In the first round of the XGBoost method, one regression tree per class is trained $\left\{T_{11},T_{12},T_{13}\right\}$. Unlike in decision trees, each regression tree produces a continuous score on the *i*th leaf.

Given a new sample to be classified, the decision rules in each tree are used to produce a set of three raw scores (one per class) $\left\{{\mathrm{rs}}_{11},{\mathrm{rs}}_{12},and{\mathrm{rs}}_{13}\right\}$, which are then transformed into probability values $\left\{p_{11},p_{12},andp_{13}\right\}$ using the SoftMax function. This in turn yields a (first-round) class prediction ${\mathrm{cp}}_{1}$ as

$$\begin{array}{cc} p_{1i} & =\frac{e^{{\mathrm{rs}}_{1i}}}{e^{{\mathrm{rs}}_{11}}+e^{{\mathrm{rs}}_{12}}+e^{{\mathrm{rs}}_{13}}},\;i=1,2,3,\\ {\mathrm{cp}}_{1} & =\underset{i=1,2,3}{\mathrm{argmax}}\left\{p_{1i}\right\}\in \left\{1,2,3\right\}. \end{array}$$

Predictions obtained in this first round are used to train the second round of trees in the forest, thereby obtaining a new regression tree per class $\left\{T_{21},T_{22},T_{23}\right\}$. After applying the decision rules in each tree, it produces a set of three raw scores $\left\{{\mathrm{rs}}_{21},{\mathrm{rs}}_{22},and{\mathrm{rs}}_{23}\right\}$. The computation of the probability per class based on the two-round forest is computed by first adding the raw score values per class, i.e., ${\mathrm{rs}}_{i}={\mathrm{rs}}_{1i}+{\mathrm{rs}}_{2i}$. Then, the SoftMax function is used to obtain the probability of class membership and a new (second-round and final) class prediction $cp_{2}$ as

$$\begin{array}{cc} p_{i} & =\frac{e^{{\mathrm{rs}}_{i}}}{e^{{\mathrm{rs}}_{1}}+e^{{\mathrm{rs}}_{2}}+e^{{\mathrm{rs}}_{3}}},\;i=1,2,3,\\ {\mathrm{cp}}_{2} & =\underset{i=1,2,3}{\mathrm{argmax}}\left\{p_{i}\right\}\in \left\{1,2,3\right\}. \end{array}$$

The same technique applies if more boosting rounds are added. If the XGBoost technique provides a forest ${\left\{T_{ji}\right\}}_{j=1,\ldots ,R,\;i=1,\ldots ,l}$ where *R* denotes the number of boosting rounds and *l* denotes the total number of classes, given a sample, the row scores per class/tree are computed ${\left\{{\mathrm{rs}}_{ji}\right\}}_{j=1,\ldots ,R,\;i=1,\ldots ,l}$ and added to obtain a final score per class ${\left\{{\mathrm{rs}}_{i}\right\}}_{i=1,\ldots ,l}$. The final score is then transformed to the probability of class membership ${\left\{p_{i}\right\}}_{i=1,\ldots ,l}$ to obtain a final class prediction cp as [45]:

(6) $$\begin{array}{cc} {\mathrm{rs}}_{i} & =\sum_{j=1}^{R}{\mathrm{rs}}_{ji},\;i=1,\ldots ,l, \end{array}$$

(7) $$\begin{array}{cc} p_{i} & =\frac{e^{{\mathrm{rs}}_{i}}}{\sum_{j=1}^{l}e^{{\mathrm{rs}}_{j}}},\;i=1,\ldots ,l, \end{array}$$

(8) $$\begin{array}{cc} \mathrm{cp} & =\underset{i=1,\ldots ,l}{\mathrm{argmax}}\left\{p_{i}\right\}\in \left\{1,\ldots ,l\right\}. \end{array}$$

Consider the specific example given in Figure 6, and a specific sample with features $F_{1}=10.24,F_{2}=3.57,F_{3}=5.02$, and $F_{4}=2.38$. In this case, the decision rules of the forest predict that this sample is associated with the leaves {2,4,2} of the first round and to the leaves {2,4,6} of the second round. Therefore, the class prediction of the sample is $C_{3}$ based on the computations listed in Table 3.

In addition to the great classification power of the XGboost method, the final classifier provides two relevant additional benefits. The first benefit is that for a given sample, the XGBoost classifier not only provides a prediction for the class of the sample, but also provides a probability-like measure of the sample belonging to each class category. From Equation (6), given a sample, the XGBoost classifier returns

- Class prediction $\mathrm{cp}=\underset{i=1,\ldots ,l}{\mathrm{argmax}}\left\{p_{i}\right\}$;
- Probability associated with the predicted class $p_{\max}=\max_{i=1,\ldots ,l}\left\{p_{i}\right\}$;
- Probability associated with each class $\left\{p_{1},p_{2},\ldots ,p_{l}\right\}$.

This additional information can be used to assess the reliability of the prediction (values of $p_{\max}$ close to 1 provide very reliable predictions) and to show the behavior of the classifier when discriminating between classes (for values of $p_{\max}$ not close to 1, the probabilities associated with each class serve as additional information on possible alternative class predictions).

The second extra benefit is that the random forest provides estimates of the feature importance automatically. The more an attribute is used to make key decisions with decision trees, the higher is its relative importance. More details can be found in ([45], Section 10.13.1).

### 4.2. XGBoost Parameters

The performance, complexity, and overfitting properties of the XGBoost method depend on the proper tuning of the hyperparameters. The best model should balance the model complexity with its predictive power. This tuning can be performed via experimentation or by using specific routines (Python scikit-learn *gridsearchCV*, for instance).

A brief description of the main XGBoost parameters used in this study is provided here. The parameters can be set into categories as shown in Table 4; it is important to highlight that in this work, any non-described parameter is set to its default value.

The first category of *general parameters* guides the overall function of the classifier. The most important parameter in this category is the *booster* parameter that defines the type of model (tree-based or linear model).

In the second category, *learning task parameters*, we find the parameters that specify the learning task and corresponding learning objective. In particular, we set the value of *objective* to be *multi:softmax* to predict the class of each data sample or *multi:softprob* to predict the probabilities of each data sample belonging to each class category, and the number of classes is defined by the parameter *num_class*. Once the probabilities are computed, the results of *multi:softmax* can be directly obtained by selecting the class with a higher probability, as shown in Section 4.1. Two relevant parameters in this category are the *seed* and *random_state* parameters. The output of the XGBoost method is a random forest, and as the name indicates, randomness is introduced in the process to avoid overfitting (for instance, when growing the trees, randomness on the selected training samples, and feature selection is introduced). Setting specific values for random seeds allow for reproducibility of the results. However, picking a convenient seed may result in over-optimistic results. Therefore, the seed should only be fixed for reproducibility and not to increase performance.

The final category corresponds to the *booster parameters* that guide the individual booster trees at each step. The three main parameters controlling the complexity of the final random forest are *n_estimators*, *max_depth*, and *learning_rate*. The *n_estimators* parameter determines the number of trees to grow per class (number of rounds); therefore, the final number of trees in the forest is n_estimators times the number of classes. The *max_depth* parameter controls the maximum depth of each decision tree. The maximum number of nodes in the forest is $n\_estimators\;\times \;num\_class\;\times \;2^{\max\_\mathrm{depth}}$. Increasing this value increases the complexity of the model and makes it more likely to overfit because it allows the model to learn very specific relations for certain samples. Finally, the *learning_rate* parameter or shrinkage parameter is analogous to the learning rate in gradient-boosted models. The learning rate corresponds to how quickly the error is corrected from each tree to the next, and it is a simple constant learning_rate∈(0,1]. The raw scores obtained in each boosting round ${\mathrm{rs}}_{ji}$ are weighted by *learning_rate* to add smaller corrections in each round; therefore, small learning rates slows down the learning and makes the boosting process more conservative. Another set of interesting parameters in this category is *reg_alpha* and *reg_lambda*, which introduce $L^{1}$ and $L^{2}$ regularization terms in the convex loss function to avoid overfitting (see [41]). Increasing these values makes the model more conservative. Finally, the parameters *subsample* and *colsample_bytree* control the fraction of items to be subsampled to train a particular tree. Every time a new tree in the random forest is trained, the algorithm selects a random sample from the complete training dataset and a random subset of the features to train the tree. The *subsample* parameter is the ratio of training instances to be selected; that is, if *subsample*=0.8 at each boosting iteration, the classifier randomly selects 80% of the training samples to train the trees in this round. Furthermore, the *colsample_bytree* parameter is the fraction of features to be sampled randomly for each tree. The default value of 1 indicates no subsampling. Lower values make the algorithm more conservative and prevent overfitting.

### 4.3. *k*-Fold Cross-Validation and Unbalanced Classification Performance Measures

It is crucial to evaluate the performance of the machine learning model on unseen data. A test dataset with new instances must be available to check the correctness of the predicted classes for evaluating the performance of a model. In multiclass classification problems (problems where each input sample must be classified into one, and only one, non-overlapping class), each sample from the test dataset has a class label that is compared to the predicted class label. A measure of correctly or incorrectly recognized classes must be defined.

Therefore, model validation has two key points:

(1) how to define the test and training datasets so that no overfitting occurs (i.e., that no too-optimistic estimates are obtained); and,
(2) how to define the performance/accuracy measure from the correctness/incorrectness of the predicted classes.

*k*-fold cross-validation is one of the most used techniques to determine the training and test data sets when a limited amount of data is available. This is because it avoids overfitting and results in a less biased or less optimistic estimate of the model skill compared to a simple train/test split [46]. In this study, a 5-fold cross-validation is used as the resampling procedure to evaluate the XGBoost model (see Figure 2).

Each fold of the cross-validation procedure provides a measure of the performance of the classification algorithms, i.e., $\left\{A_{1},A_{2},\ldots ,A_{5}\right\}$; the total predicted performance is given by its mean

$$\begin{array}{c} \bar{A}=\left(A_{1}+A_{2}+\cdots +A_{5}\right)/5. \end{array}$$

The standard deviation of these performance measures can also be computed as

$$\begin{array}{c} \sigma_{\bar{A}}=\sqrt{\sum_{i=1}^{5}{\left(A_{i}-\bar{A}\right)}^{2}/5}. \end{array}$$

A large standard deviation $\sigma_{\bar{A}}$ indicates that the performance measures $A_{i},\;i=1,\ldots ,5$ are far from its mean $\bar{A}$. This suggests that samples were not selected appropriately or that the method was too subsample-dependent.

In general, the classification performance for a given fold is defined by specific measures of the confusion matrices (see details below). Although this is not the approach used in this work, it is common practice to obtain the total performance by first adding the five confusion matrices associated with each fold directly and then computing the performance of the total matrix, despite the performance measures being nonlinear.

Specific performance measures used in the present work are described as follows: Given the classification results associated with a fold, the correctness of the classification method associated with this fold is evaluated by first computing the number of correctly recognized class samples (true positives, TP), the number of correctly recognized samples that do not belong to the class (true negatives, TN), and samples that either were incorrectly assigned to a class (false positives, FP) or that were not recognized as class samples (false negatives, FN). This information is summarized in a multiclass confusion matrix [7,47]. Indeed, let $\left\{C_{1},C_{2},C_{3},C_{4},C_{5}\right\}$ denote the five class labels associated with the experiment (undamaged, damage 1, damage 2, damage 3, and damage 4, respectively), and $C_{ij}$ denote the number of samples belonging to class $C_{i}$, which have been classified as belonging to class $C_{j}$. This information can be stored in the confusion matrix listed in Table 5.

Then, for a given class $C_{i}$, we denote by ${\mathrm{tp}}_{i}$, ${\mathrm{tn}}_{i}$, ${\mathrm{fn}}_{i}$, and ${\mathrm{fp}}_{i}$, the number of samples that, with respect to class $C_{i}$, are TPs, TNs, FNs, and FPs, respectively; these are computed from the confusion matrix as

$$\begin{array}{c} {\mathrm{tp}}_{i}=C_{ii},\;{\mathrm{tn}}_{i}=\sum_{\underset{k\neq i}{k=1}}^{l}\sum_{\underset{j\neq i}{j=1}}^{l}C_{kj},\;{\mathrm{fn}}_{i}=\sum_{\underset{j\neq i}{j=1}}^{l}C_{ij},\;{\mathrm{fp}}_{i}=\sum_{\begin{array}{c} k=1\\ k\neq i \end{array}}^{l}C_{ki}, \end{array}$$

where *l* denotes the total number of classes or labels, i.e., l=5 in the present work. In addition, following [47], we introduce the performance measures associated with class $C_{i}$ as

$$\begin{array}{cc} {\mathrm{accuracy}}_{i} & =\frac{{\mathrm{tp}}_{i}+{\mathrm{tn}}_{i}}{{\mathrm{tp}}_{i}+{\mathrm{tn}}_{i}+{\mathrm{fn}}_{i}+{\mathrm{fp}}_{i}},\\ {\mathrm{precision}}_{i} & =\frac{{\mathrm{tp}}_{i}}{{\mathrm{tp}}_{i}+{\mathrm{fp}}_{i}},\\ {\mathrm{recall}}_{i} & =\frac{{\mathrm{tp}}_{i}}{{\mathrm{tp}}_{i}+{\mathrm{fn}}_{i}},\\ {\mathrm{specificity}}_{i} & =\frac{{\mathrm{tn}}_{i}}{{\mathrm{tn}}_{i}+{\mathrm{fp}}_{i}}, \end{array}$$

and

$$\begin{array}{cc} F{}_{1}{-\mathrm{score}}_{i} & =2\frac{{\mathrm{Precision}}_{i}\times {\mathrm{recall}}_{i}}{{\mathrm{precision}}_{i}+{\mathrm{recall}}_{i}}=\frac{2{\mathrm{tp}}_{i}}{2{\mathrm{tp}}_{i}+{\mathrm{fn}}_{i}+{\mathrm{fp}}_{i}}, \end{array}$$

where ${\mathrm{tp}}_{i}+{\mathrm{tn}}_{i}+{\mathrm{fn}}_{i}+{\mathrm{fp}}_{i}$ coincides with the total number of tested samples (n/5=1148 for a specific fold or n=5740 if the global added confusion matrix is considered).

The quality of the classification strategy for the fold is assessed in this case because of data imbalance, which uses macro-averaging global performance measures that treat all classes equally instead of favoring the larger ones. Global measures are computed by averaging the measures obtained in each class, namely

$$\begin{array}{cc} \mathrm{accuracy} & =\frac{1}{l}\sum_{i=1}^{l}{\mathrm{accuracy}}_{i},\\ \mathrm{precision} & =\frac{1}{l}\sum_{i=1}^{l}{\mathrm{precision}}_{i},\\ \mathrm{recall} & =\frac{1}{l}\sum_{i=1}^{l}{\mathrm{recall}}_{i},\\ \mathrm{specificity} & =\frac{1}{l}\sum_{i=1}^{l}{\mathrm{specificity}}_{i},\\ F{}_{1}-\mathrm{score} & =2\frac{\mathrm{precision}\times \mathrm{recall}}{\mathrm{precision}+\mathrm{recall}}, \end{array}$$

the global F${}_{1}$-score measure is not computed by averaging the per-class F${}_{1}$-score measures but by using the global precision and recall measures.

The final overall performance measure of the classifier is obtained by computing the average and standard deviation of the five-fold performance measures.

## 5. Proposed Methodology: Real-Time Structural Damage Diagnose

Section 3 and Section 4 describe the training and validation of the XGBoost classifier using the samples obtained from the experiments under different white noise signals and different structural states. However, the described strategy can be used for real-time damage detection. Given a new sample associated with a specific wind turbine, a fast real-time prediction of the structural state of the structure can be performed.

**Figure 7:**
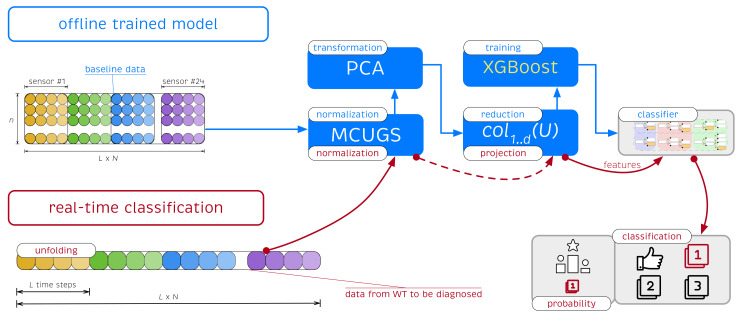
Flowchart of the proposed methodology of real-time structural damage diagnosis to classify a new wind turbine (WT). Data from a structure were first normalized and then projected into the PCA model. Finally, XGBoost is applied to obtain a class prediction and probability of class membership.

In this context, an offline strategy is adopted. In the offline stage, the *baseline* data (set of initial samples used to generate the XGBoost classifier) was used to determine the pre-trained XGBoost classifier. The offline stage stores the MCUGS normalization parameters, the PCA projection matrix selects the relevant features, and the classifier is given in the form of a forest regression tree. This information is then used in the online stage for real-time classification of a new observation. A flowchart of the proposed approach to illustrate how the SHM strategy is applied when a new wind turbine (WT) should be classified is depicted in Figure 7.

Given a new single observation of the wind turbine to be diagnosed that contains signals measured by the N=24 sensors during the L=2417 time instants, we construct a new data row vector $z^{⊤}$ unfolded as any of the rows of matrix X in Equation (4), i.e.,

$$\begin{array}{c} z^{⊤}=\left(z_{1}^{1},z_{2}^{1},\ldots ,z_{L}^{1},z_{1}^{2},z_{2}^{2},\ldots ,z_{L}^{2},\ldots ,z_{1}^{N},z_{2}^{N},\ldots ,z_{L}^{N}\right). \end{array}$$

The collected data were first normalized using the pre-stored MCUGS parameters (the mean $\mu_{j}^{k}$ and the standard deviation $\sigma_{\mathrm{MCUGS}}^{k}$ given in Equations (1) and (2), respectively), which produced the normalized raw vector ${\overset{˘}{z}}^{⊤}$ defined as

$$\begin{array}{c} {\overset{˘}{z}}_{j}^{k}=\frac{z_{j}^{k}-\mu_{j}^{k}}{\sigma_{\mathrm{MCUGS}}^{k}},\;k=1,\ldots ,N,\;j=1,\ldots ,L. \end{array}$$

The normalized data were then projected using the pre-stored PCA projection matrix to select the relevant features to be used in the XGBoost classifier. ${\overset{˘}{z}}^{⊤}$ is projected onto the vector space spanned by the first *d* principal components stored in the matrix ${\mathrm{col}}_{1..d}\left(U\right)$ using the vector-to-matrix product

$$\begin{array}{c} y^{⊤}={\overset{˘}{z}}^{⊤}{\mathrm{col}}_{1..d}\left(U\right)\in R^{d}. \end{array}$$

$y^{⊤}$ is a *d*-dimensional vector that is the projection of $z^{⊤}$ into the PCA model. The components of this vector are the *d* features $F_{j}=y^{⊤}e_{j},\;j=1,\ldots ,d$, which are the inputs of the XGBoost classifier; see Equation (5).

As shown in Section 4.1 and in Table 3, features $F_{j}$ associated with the wind turbine to be diagnosed are directly inserted into the *forest* classifier, which returns a set of probabilities ${\left\{p_{i}\right\}}_{i=1,\ldots ,l}$, where *l* denotes the total number of different structural states and $p_{i}$ denotes the probability that the sample belongs to the structural state *i*. Thus, the real-time classification strategy provides the following: (1) the structural state/class prediction cp; (2) the related probability of class membership $p_{\max}$ used for reliability (we can associate a higher or lower level of confidence in our decision based on the value of $p_{\max}$); and (3) probability associated with each of the other structural states.

The problem considered in the present work is related to real-time structural damage diagnosis and classification. A closely related problem is the early detection of incipient damage (prognosis). Prognosis methodologies contribute a predictive maintenance option that provide the decision-maker the flexibility to determine whether and when to act before the structure is severely damaged [48]. To this goal, instead of a structural damage classification problem, anomaly detection can be considered. In the case of anomaly detection, a similar approach can be used to classify the structure as healthy or not healthy.

## 6. Results

The results of damage detection and classification for small-scale jacket-type wind turbines are presented in this section. More precisely, Section 6.1 shows the time-history raw signals measured by the accelerometers and the corresponding normalized signals. Section 6.1 emphasizes the existing differences between MCGS and MCUGS. The MCUGS normalization technique is a novel technique introduced for the first time in this study. Section 6.2 describes the data transformation and data reduction using PCA results, which are key steps prior to the final classification. A detailed analysis of the capabilities and limitations of PCA to discriminate between different structural states are presented in Section 6.2. Finally, the results of damage detection and classification in terms of the confusion matrices and the averaged performance measures are presented in Section 6.3, along with a reliability study of the classification results.

### 6.1. Data Normalization: Raw Signal vs. MCUGS Signal

**Figure 8:**
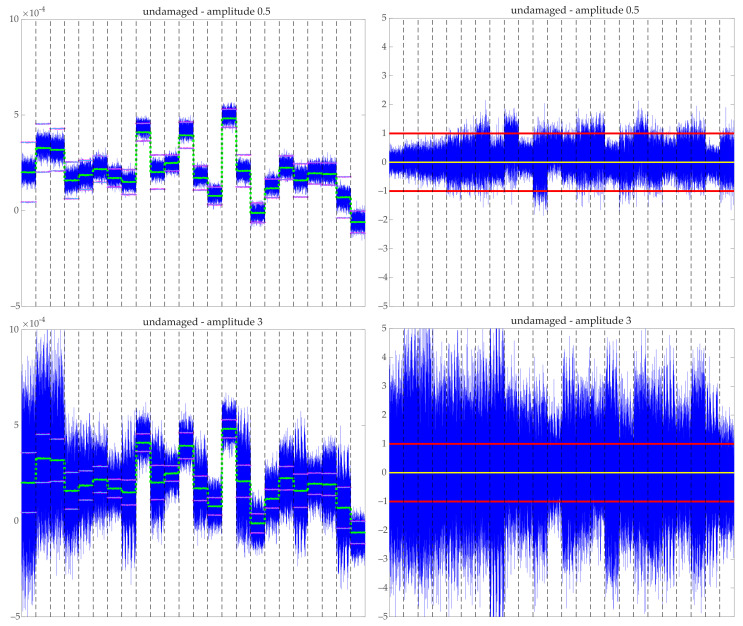
Raw signal (left) versus MCUGS signal (right) for two undamaged samples (amplitudes 0.5 (top) and 3 (bottom). Raw signals (blue) are shown along with the mean values $\mu_{j}^{k}$ (green) and the representation of the standard deviations $\mu_{j}^{k}\pm \sigma_{\mathrm{MCUGS}}^{k}$ (cyan) and $\mu_{j}^{k}\pm \sigma_{\mathrm{MCGS}}^{k}$ (magenta). The normalized data have zero mean (yellow) and unitary deviation (red). Dashed vertical lines separate the measures of the 24 sensors.

**Figure 9:**
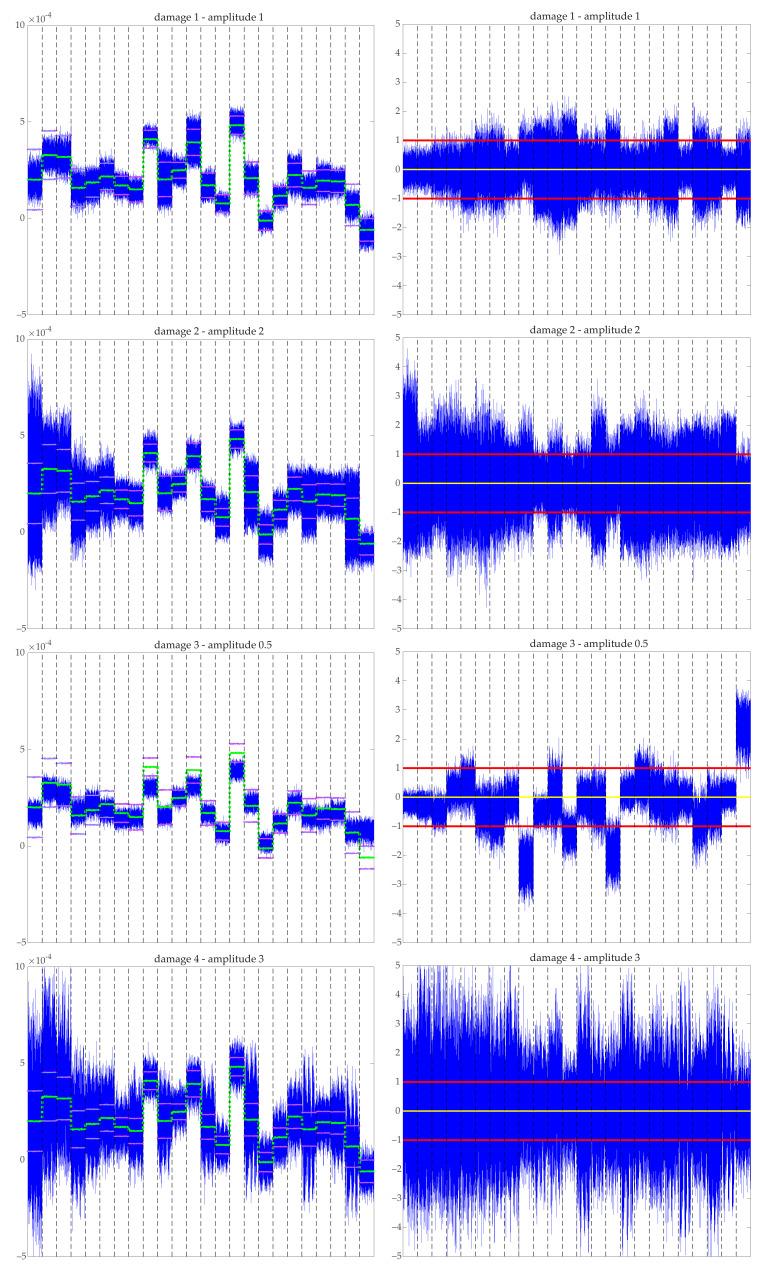
Raw signal (left) versus MCUGS signal (right) for four damaged samples. Raw signals (blue) are shown along with the mean values $\mu_{j}^{k}$ (green) and the representation of the standard deviations $\mu_{j}^{k}\pm \sigma_{\mathrm{MCUGS}}^{k}$ (cyan) and $\mu_{j}^{k}\pm \sigma_{\mathrm{MCGS}}^{k}$ (magenta). The normalized data have zero mean (yellow) and unitary deviation (red). Dashed vertical lines separate the measures of the 24 sensors.

Figure 8 and Figure 9 show random samples of data collected in matrix X (see Equation (3)) before and after normalization. Two samples associated with the undamaged structure with minimum and maximum amplitudes are shown in Figure 8.

Raw unpreprocessed data containing the values of the acceleration over time for each sensor (recall that the values per sensor are placed one after another with a total of 24 blocks) are shown along with its associated normalized MCUGS signal. The MCGS normalized signal is not seen because it is nearly equal to the MCUGS signal. The raw data are shown along with the time evolution of the mean value of all samples per time and sensor $\mu_{j}^{k}$. The mean value is nearly a piecewise-constant function (nearly constant per sensor), i.e., $\mu_{j}^{k}\approx \mu^{k}$; therefore, the MCGS and MCUGS provide nearly identical normalized data. In addition, the values of the standard deviation of the raw data, shown in the graphics as $\mu_{j}^{k}\pm \sigma_{\mathrm{MCUGS}}^{k}$ and $\mu_{j}^{k}\pm \sigma_{\mathrm{MCGS}}^{k}$, are obtained. Because $\mu_{j}^{k}\approx \mu^{k}$, the values for the MCGS and MCUGS standard deviations are nearly indistinguishable. The resulting MCUGS normalized data have zero mean, and both unitary block and global deviation. The same information is shown in Figure 9 for a random sample of each damaged structure. For the damaged structures, the mean value is nearly a piecewise-constant function; therefore, the MCGS and MCUGS behave similarly. At first sight, only the normalized data for the third type of damage seem to behave differently.

### 6.2. Data Transformation and Data Reduction: Feature Extraction

**Figure 10:**
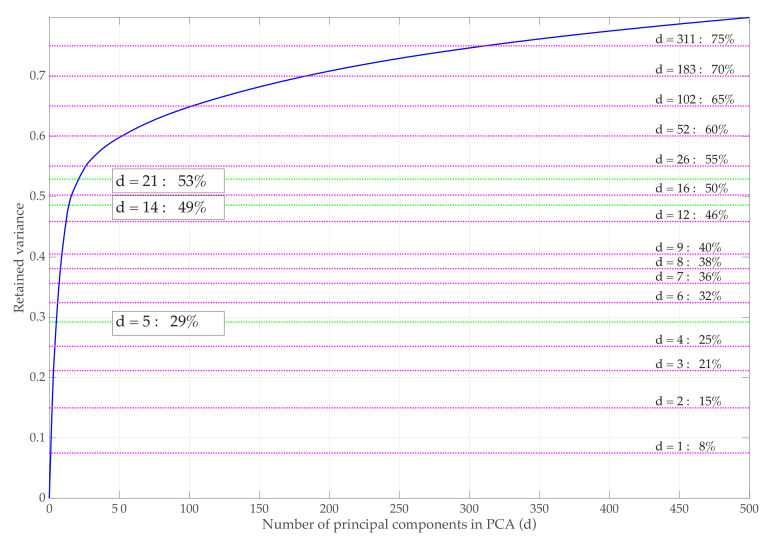
Cumulative explained variance of the PCA model when varying the number of principal components. *d* = 5, *d* = 14 and d=21 components account for 29%,49%, and 53% of the variance.

The PCA is applied to the data matrix $X\in R^{n\times D}$ in Equation (3), where the number of samples is n=5470 and the number of features is D= 58,008, to select the *d* most relevant features (*d* principal components). Figure 10 shows the cumulative explained variance retained by the PCA method, i.e., $\sum_{i=1}^{d}\lambda_{i}/\sum_{i=1}^{D}\lambda_{i}$ when varying the values of *d*, thereby characterizing the retained linear manifold.

The number of principal components needed to retain a relevant amount of variance is considerably high (d=311 components are required to account for 75% of the variance). However, we show that the use of the XGBoost classifier allows the accurate classification of data, thereby considering only very few components of the PCA decomposition.

**Figure 11:**
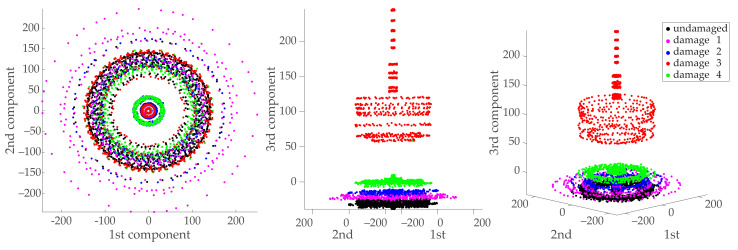
Projection onto the first three principal components of the data in matrix X: first two principal components (**left**), third principal component (**middle**), and three-dimensional view (**right**) of the first three principal components.

Figure 11 shows the first three PCA components of the three-dimensional manifold computed from the initial normalized MCUGS data as

$$Y=X\;{\mathrm{col}}_{1..3}\left(U\right).$$

The first two components distribute the samples in a radial configuration; different radii represent different excitation amplitudes—, where the undamaged and damaged samples cannot be clearly separated. The third component clearly separates the samples from the third damaged structure, and it distributes the other samples at different heights.

**Figure 12:**
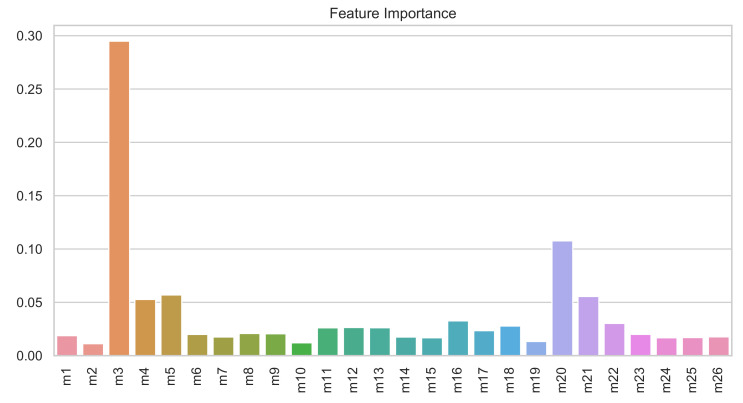
Relative feature importance of the 26 first principal components in the XGBoost model.

The benefit of using the XGBoost classifier is that the ensembles of decision trees also provide an estimate of how useful or valuable each feature is in the construction of the boosted decision trees within the model in addition to it being a very competitive technique to classify the data. Figure 12 shows the feature importance of the first 26 principal components.

**Figure 13:**
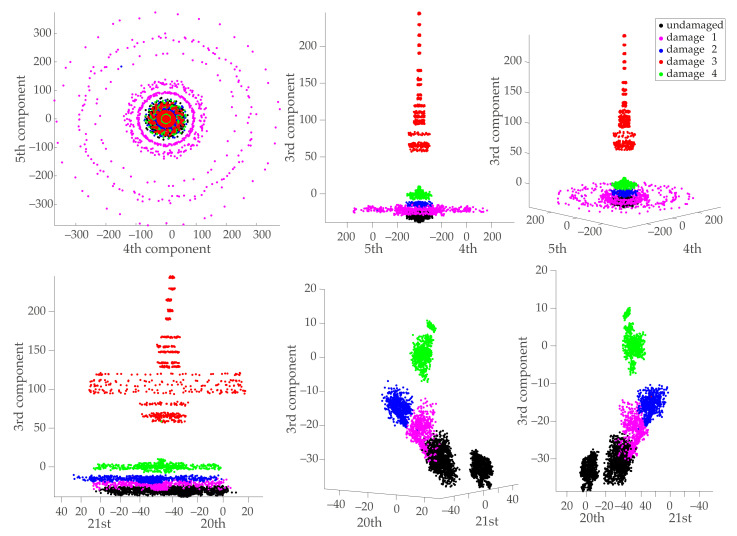
Projection onto relevant principal components of the data in matrix X in Equation (3): 4th and 5th principal components (**top**) and 20th and 21st principal components (**bottom**). The two figures on the bottom right are zoomed in.

The most relevant feature for this problem is the third PCA component, followed by the 4th, 5th, 20th, and 21st. Figure 13 shows the most relevant PCA components selected by the XGBoost classifier. The first principal components are not always the best option for detecting and classifying damage, as suggested by Mujica et al. [49]. In this case, when compared to Figure 11, the 4th and 5th or the 20th and 21st components seem to better separate the data. Furthermore, once the relevant features are depicted, the most challenging separation task is to classify the samples associated with undamaged and damaged 1-type structures appropriately.

### 6.3. Extreme Gradient Boosting and 5-Fold Cross-Validation

Once the relevant features are extracted using PCA, the transformed data

$$Y_{d}=X\;{\mathrm{col}}_{1..d}\left(U\right)\in R^{n\times d}$$

were used as the input to the 5-fold cross-validation procedure to train and test the XGBoost classifier random forests. As described in Figure 2, the data are first subdivided into five folds, and five separate XGBoost classifiers are trained and tested using the corresponding subsamples. The parameters of the XGBoost classifier were tuned using Python scikit-learn *gridsearchCV* to achieve a certain compromise between performance results and model complexity. Increasing the complexity of the random forests (e.g., larger values of *n_estimators* or *m_depth*) yields more accurate classification results; however, it generates overfitted models. The optimal XGBoost parameters used to train all models are summarized in Table 6.

**Figure 14:**
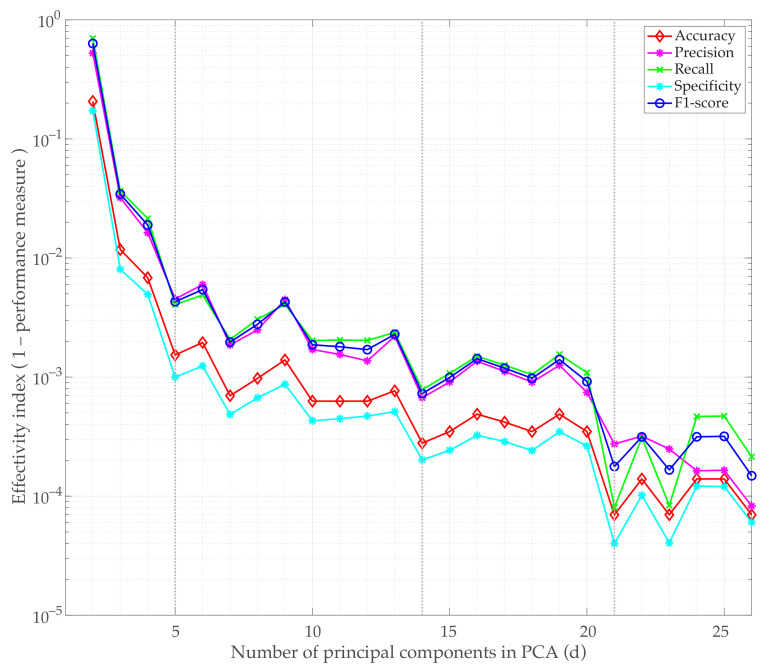
Averaged performance metrics versus the number of retained principal components in the PCA model (semi-logarithmic plot in the vertical axis). The vertical axis represents the effectivity index, i.e., one minus the performance measure.

Figure 14 shows the performance measures obtained by averaging the performance measures (accuracy, precision, recall, specificity, and $F_{1}$-score) of each fold when varying the retained number of principal components (*d*) used as input feature matrix for the XGBoost. The effectivity index associated with each measure $\bar{A}\in \left[0,1\right]$, defined as $\rho =1-\bar{A}$, is depicted. Since a perfect classification corresponds to $\bar{A}=1$, the best results are obtained when ρ is close to zero. A jump in the quality of the classifier is obtained when using up to 5 and 21 principal components as input features, which is in good agreement with the feature importance measures provided by the XGBoost classifier (see Figure 12). In addition, taking d=14 provides very good results.

Specific performance measures $\bar{A}\pm \sigma_{\bar{A}}$ obtained for d=5,d=14, and d=21 are listed in Table 7.

Very good performance metrics are obtained even when only 5 features are used to classify the samples. For values larger than or equal to 21 principal components, a nearly perfect classification is obtained. For the parameters described in Table 6, considering d=21 yields only three misclassified samples out of 5470, while for d=27, a perfect classification is obtained.

Table 8, Table 9 and Table 10 summarize the total added confusion matrices associated with the three specific values d=5,14, and 21.

The confusion matrices confirm the observations of most relevant XGBoost features of the data in Figure 13. The third and fourth types of damaged samples (labels *damage 3* and *damage 4*) are easily separated from the other samples. These samples are perfectly classified in three cases: d=5,d=14, and d=21. Increasing the number of features allows for better separation of the second type of damage. For d=21, only one undamaged sample and two damaged 1 samples are incorrectly classified.

The small-scale jacket-type wind turbine is a benchmark structure for testing, validating, and comparing different damage-detection approaches. Vidal et al. [7] used a combination of PCA and quadratic SVM for damage classification of the same structure and the same types of damage. The same benchmark structure was considered in [13,14], but with different types of damage. The overall performance of these approaches is excellent. However, in comparison to the approach in the present work, two points must be considered.

- Vidal et al. [7] consider 443 principal components that account for 85% of the variance, while we consider 5,14 or 21 principal components. This represents a data reduction (leading to reduced memory requirements) of 98.87%,96.84%, or 95.26%, respectively. The extent of the data reduction is even more significant if we compare the 5,14 or 21 principal components with respect to the 58,008 columns in matrix X in Equation (3). In this case, it represents a data reduction of 99.99%,99.98% or 99.96%, respectively.
- In this study, when d=5 and d=14 principal components have been considered, a perfect classification is obtained for damage 3 and damage 4. Damage 2 is also classified perfectly when d=21. However, the only perfectly classified structural state in [7] is damage 4.

The confusion matrices presented in Table 8, Table 9 and Table 10 summarize the overall performance of the proposed approach with respect to the actual and predicted classes. However, one of the benefits of the XGBoost classifier is that it provides a probability-like measure of the sample belonging to this predicted class $p_{\max}$ along with the probabilities associated with the other class membership ${\left\{p_{i}\right\}}_{i=1,\ldots ,5}$. Therefore, the reliability of the prediction can be assessed based on these probabilities.

**Figure 15:**
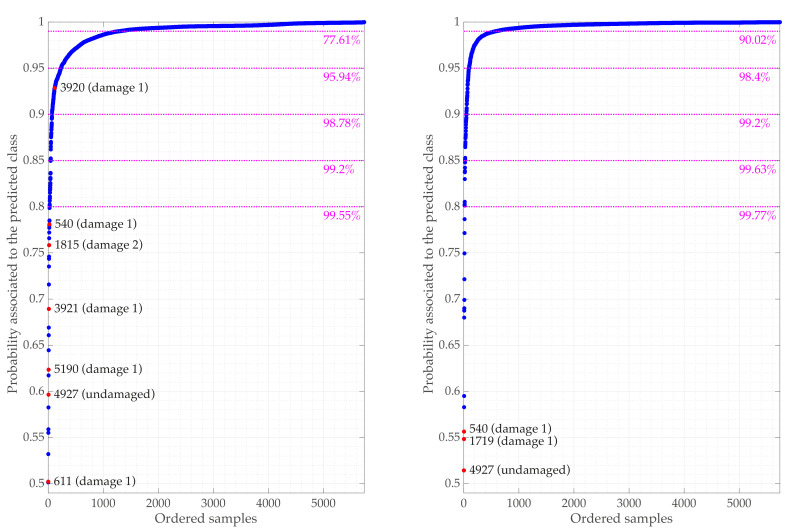
Probability associated with the predicted class $p_{\max}$ (d=14 in the left and d=21 in the right) for all samples ordered in the ascending order of probability $p_{\max}$. The misclassified samples are represented in red along with the sample number and actual class tags.

Figure 15 depicts a probability-like measure associated with the predicted class $p_{\max}$ in cases d=14 and d=21, for all 5470 samples (ordered in ascending order of probability). The misclassified samples (7 and 4 respectively) are highlighted in red. A high level of confidence is assigned to most classification results considering d=14 and d=21 features to classify the samples. 95.94% and 98.4% of the samples are classified with a probability larger or equal than 0.95, respectively. Furthermore, all but one of the misclassifications are associated with probabilities smaller than 0.8. In fact, in the case d=21, the three misclassified samples are associated with the three smaller probabilities. Furthermore, for d=21, 99.77% of the samples were (correctly) classified with a probability greater than or equal to 80%. Similarly, 90.02% of the samples were classified with a probability greater than or equal to 99%.

**Figure 16:**
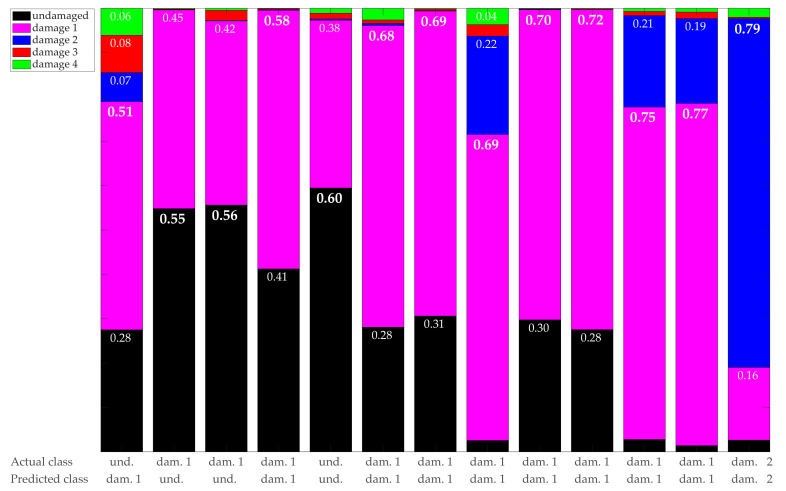
Probability distribution ${\left\{p_{i}\right\}}_{i=1,\ldots ,5}$ for samples with $p_{\max}<0.8$ in the case d=21 (in the increasing order of $p_{\max}$). The first three columns are associated with the three samples that are incorrectly classified.

As a complement to Figure 15, Figure 16 depicts the probability distribution for the 13 samples with smaller assigned probabilities, $p_{\max}<0.8$. The first three columns are associated with the three samples that are incorrectly classified (sample tags 4927, 1719, and 540, respectively). These samples coincide with the samples with the minimum values of $p_{\max}$. Although the class prediction is incorrect in these three cases, the actual class is associated with the second maximum probability.

- The sample in the first column (undamaged) is wrongly classified as damage 1 with a $p_{\max}=51\%$, while the probability associated with the actual class is $p_{1}=28\%$.
- The sample in the second column (damage 1) is wrongly classified as undamaged with a $p_{\max}=55\%$, while the probability associated with the actual class is $p_{2}=45\%$.
- The sample in the third column (damage 1) is wrongly classified as undamaged with a $p_{\max}=56\%$, while the probability associated with the actual class is $p_{2}=42\%$.
- Nine columns (1,2,3,4,5,6,7,9, and 10) are associated with samples where the classifier detects both characteristics of the undamaged and damage 1 structures and manages to detect the predominant class in 6 of the nine cases.
- Four of the columns (8,11,12, and 13) are associated with samples where the classifier detects both characteristics of damage 1 and damage 2 structures, even though it correctly detects the predominant class (correct prediction) in all cases.

## 7. Conclusions and Future Work

A vibration-response-only damage detection and classification methodology was proposed for the SHM of supporting structures of offshore wind turbines. The approach was tested on a small-scale laboratory setup with five different structural states: an undamaged structure and a 5 mm crack at four different bars. The developed SHM methodology that combines data preprocessing, a dimensionality reduction via PCA and a machine learning classification stage through XGBoost method has a remarkable performance, even for a small retained cumulative proportion of variance.

The main conclusions of this work are:

- Data Preprocessing: includes data acquisition, data normalization (using MCUGS for the first time) taking into account the differences in magnitude obtained by the 8 triaxial accelerometers attached to the jacket-type wind-turbine foundation, and data unfolding.
- Data transformation: a dimensionality reduction stage is applied to perform feature extraction and reduce the bid data obtained with the sensors using PCA. The selection of the number of principal components is done according to the average classification accuracy obtained with the methodology. The best results were obtained when considering 21 principal components as relevant features, which only account for 53% of the variance and represents a 99.96% of data reduction.
- Damage Detection and Classification: the machine learning model is designed to define XGBoost hyperparameters (general parameters, learning task parameters, and booster parameters). These parameters play a fundamental role in improving the model performance while avoiding overfitting. The XGBoost classifier manages to classify nearly all samples appropriately (only three out of 5470 samples are misclassified).
- The estimation of class membership probabilities allows the assignment of a high level of confidence to most results (only 13 samples are classified with a probability smaller than 0.8, and 98% of the samples are classified with a probability larger than 0.95). This provides unambiguous results. For samples in which a clear classification is not obtained, the probability distribution provides a better insight into the type of structure associated with the sample (usually two classes are indicated by the classifier as possible predictions).
- Online Monitoring: the proposed methodology can process new data using the MCUGS normalization parameters, the PCA projection matrix and the XGBoost classifier for real-time classification of a new observation.

Future work is expected to focus on two main areas. Nonlinear manifold learning techniques will be explored to increase the separability capacity of the dimensionality reduction stage before entering the classifier; further, the possibility of combining both linear and nonlinear feature extraction techniques will be studied. This research line is addressed to better identify low-dimensional manifolds present when the data include nonlinear behaviors. The developed damage classification methodology will be tested in more complex and realistic structures.

## Figures and Tables

**Table 1:** Description of the eight sensors location in the structure.

Sensor ID	Location
1	Joint between the shaker and nacelle top beam
2	Joint between the nacelle and tower
3	Joint between middle and bottom tubular section of the tower
4	Joint between the four bars in the upper left side of the jacket
5	Joint between the four bars in the lower left side of the jacket
6	Joint between the four bars in the upper central side of the jacket
7	Joint between the four bars in the lower right side of the jacket
8	Joint between the four bars in the lower central side of the jacket

**Table 2:** Labels, number of tests per amplitude and total number of tests for each structure.

Structural State (Class)	Number of Tests (per Amplitude)	Total Number of Tests (4 Amplitudes)
undamaged	615	2460
damage 1	205	820
damage 2	205	820
damage 3	205	820
damage 4	205	820
5740		

**Table 3:** Classification of the sample with features $F_{1}=10.24,F_{2}=3.57,F_{3}=5.02$ and $F_{4}=2.38$ based on a two-round forest (Figure 6). The final score per class is then transformed to probability of class membership to obtain a final class prediction (cell with gray background).

First-Round Scores	Second-Round Scores	Final Scores per Class	Final Probabilities
${\mathrm{rs}}_{11}=-0.22$	${\mathrm{rs}}_{21}=-0.20$	${\mathrm{rs}}_{1}=-0.42$	$p_{1}=0.20$
${\mathrm{rs}}_{12}=-0.20$	${\mathrm{rs}}_{22}=-0.15$	${\mathrm{rs}}_{2}=-0.35$	$p_{2}=0.21$
${\mathrm{rs}}_{13}=0.40$	${\mathrm{rs}}_{23}=0.29$	${\mathrm{rs}}_{3}=0.69$	$p_{3}=0.59$

**Table 4:** Description of the key parameters used for XGBoost classification.

Parameter	Value	Default	Description
30General Parameters			
booster	gbtree		type of learner (tree-based model)
30Learning Task Parameters			
objective	multi:softmax		multiclass classification/predicted class
	multi:softprob		multiclass classification/predicted probabilities
num_class	N		number of classes
seed	Z		seed used to generate the folds (reproducibility)
random_state	Z		seed for the random number generator (reproducibility)
30Booster Parameters			
n_estimators	N	100	number of trees grown per class (rounds)
max_depth	N	none	maximum depth of the trained decision trees
learning_rate	(0,1]	0.3	learning rate or shrinkage parameter
reg_alpha	[0,∞)	0	$L^{1}$ regularization in the objective loss function
reg_lambda	[0,∞)	1	$L^{2}$ regularization in the objective loss function
subsample	(0,1]	1	fraction of observations to be subsampled
colsample_bytree	(0,1]	1	fraction of features to be subsampled

**Table 5:** Multiclass confusion matrix. The colored cells correspond to the true positive (green), true negatives (cyan), false negatives (orange) and false positives (magenta) associated with the $C_{4}$ class.

		Predicted Class				
		Class $C_{1}$	Class $C_{2}$	Class $C_{3}$	Class $C_{4}$	Class $C_{5}$
Actual Class	Class $C_{1}$	$C_{11}$	$C_{12}$	$C_{13}$	$C_{14}$	$C_{15}$
	Class $C_{2}$	$C_{21}$	$C_{22}$	$C_{23}$	$C_{24}$	$C_{25}$
	Class $C_{3}$	$C_{31}$	$C_{32}$	$C_{33}$	$C_{34}$	$C_{35}$
	Class $C_{4}$	$C_{41}$	$C_{42}$	$C_{43}$	$C_{44}$	$C_{45}$
	Class $C_{5}$	$C_{51}$	$C_{52}$	$C_{53}$	$C_{54}$	$C_{55}$

**Table 6:** XGBoost parameters.

Parameter	Value
booster	gbtree
objective	multi:softmax/multi:softprob
num_class	5
seed	27
random_state	42
n_estimators	200
max_depth	3
learning_rate	0.1
reg_alpha	0.1
reg_lambda	1
subsample	0.7
colsample_bytree	0.3

**Table 7:** Averaged performance measures of the XGBoost classifier when varying the number of principal components *d* along with its associated standard deviation.

	5 PCA Components	14 PCA Components	21 PCA Components
Accuracy	0.99860627±0.00049276	0.99951219±0.00027875	0.99979094±0.00017070
Precision	0.99614306±0.00139273	0.99875525±0.00085294	0.99959326±0.00045093
Specificity	0.99906633±0.00030626	0.99965960±0.00021238	0.99983739±0.00013901
Recall	0.99620531±0.00149676	0.99847088±0.00094042	0.99943089±0.00058063
F${}_{1}$-score	0.99617413±0.00142646	0.99861287±0.00079226	0.99951207±0.00040777

**Table 8:** Total added confusion matrix for d=5. Correct decisions are placed in cells with green background while wrong decisions are placed in cells with red background.

		Predicted Class				
		Undamaged	Damage 1	Damage 2	Damage 3	Damage 4
Actual Class	undamaged	2454	6	0	0	0
	damage 1	6	807	7	0	0
	damage 2	0	1	819	0	0
	damage 3	0	0	0	820	0
	damage 4	0	0	0	0	820

**Table 9:** Total added confusion matrix for d=14. Correct decisions are placed in cells with green background while wrong decisions are placed in cells with red background.

		Predicted Class				
		Undamaged	Damage 1	Damage 2	Damage 3	Damage 4
Actual Class	undamaged	2459	1	0	0	0
	damage 1	3	815	2	0	0
	damage 2	0	1	819	0	0
	damage 3	0	0	0	820	0
	damage 4	0	0	0	0	820

**Table 10:** Total added confusion matrix for d=21. Correct decisions are placed in cells with green background while wrong decisions are placed in cells with red background.

		Predicted Class				
		Undamaged	Damage 1	Damage 2	Damage 3	Damage 4
Actual Class	undamaged	2459	1	0	0	0
	damage 1	2	818	0	0	0
	damage 2	0	0	820	0	0
	damage 3	0	0	0	820	0
	damage 4	0	0	0	0	820

## Data Availability

The data presented in this study are available on request from the corresponding author.

## Abbreviations

The following abbreviations are used in this manuscript:

AIS	Artificial immune systems
CNN	Convolutional neural networks
EOC	Environmental and operational conditions
fn	False negative
fp	False positive
*k*NN	*k* nearest neighbors
MCGS	Mean-centered group scaling
MCUGS	Mean-centered unitary group scaling
ML	Machine learning
O&M	Operations and maintenance
PCA	Principal component analysis
RNN	Recurrent neural networks
RMS	Root mean square
SEAMAC	Sensor elimination by modal assurance criterion
SHM	Structural health monitoring
SOM	Self-organizing maps
SVM	Support vector machines
tn	True negative
tp	True positive
*t*-SNE	*t*-distributed stochastic neighbor embedding
WT	Wind turbine
XGBoost	Extreme gradient boosting

## References

[1] Fritzen C.P., Kraemer P., Klinkov M. (2011). An integrated SHM approach for offshore wind energy plants. Structural Dynamics.

[2] Weng S., Zhu H., Xia Y., Li J., Tian W. (2020). A review on dynamic substructuring methods for model updating and damage detection of large-scale structures. Adv. Struct. Eng..

[3] Huang M., Cheng X., Lei Y. (2021). Structural damage identification based on substructure method and improved whale optimization algorithm. J. Civ. Struct. Health Monit..

[4] Tibaduiza Burgos D.A., Anaya Vejar M., Pozo F. (2020). Pattern Recognition Applications in Engineering.

[5] Lian J., Cai O., Dong X., Jiang Q., Zhao Y. (2019). Health monitoring and safety evaluation of the offshore wind turbine structure: A review and discussion of future development. Sustainability.

[6] Tziavos N.I., Hemida H., Dirar S., Papaelias M., Metje N., Baniotopoulos C. (2020). Structural health monitoring of grouted connections for offshore wind turbines by means of acoustic emission: An experimental study. Renew. Energy.

[7] Vidal Y., Aquino G., Pozo F., Gutiérrez-Arias J.E.M. (2020). Structural Health Monitoring for Jacket-Type Offshore Wind Turbines: Experimental Proof of Concept. Sensors.

[8] Tsiapoki S., Bahrami O., Häckell M.W., Lynch J.P., Rolfes R. (2020). Combination of damage feature decisions with adaptive boosting for improving the detection performance of a structural health monitoring framework: Validation on an operating wind turbine. Struct. Health Monit..

[9] Jondral F.K. (2018). White Gaussian Noise—Models for Engineers. Frequenz.

[10] Huang M., Lei Y., Li X., Gu J. (2021). Damage Identification of Bridge Structures Considering Temperature Variations-Based SVM and MFO. J. Aerosp. Eng..

[11] Gu J., Gul M., Wu X. (2017). Damage detection under varying temperature using artificial neural networks. Struct. Control. Health Monit..

[12] Azimi M., Eslamlou A.D., Pekcan G. (2020). Data-driven structural health monitoring and damage detection through deep learning: State-of-the-art review. Sensors.

[13] Puruncajas B., Vidal Y., Tutivén C. (2020). Vibration-Response-Only Structural Health Monitoring for Offshore Wind Turbine Jacket Foundations via Convolutional Neural Networks. Sensors.

[14] Hoxha E., Vidal Y., Pozo F. (2020). Damage Diagnosis for Offshore Wind Turbine Foundations Based on the Fractal Dimension. Appl. Sci..

[15] Khadka A., Fick B., Afshar A., Tavakoli M., Baqersad J. (2020). Non-contact vibration monitoring of rotating wind turbines using a semi-autonomous UAV. Mech. Syst. Signal Process..

[16] Papadimitriou C., Fritzen C.P., Kraemer P., Ntotsios E. (2011). Fatigue predictions in entire body of metallic structures from a limited number of vibration sensors using Kalman filtering. Struct. Control. Health Monit..

[17] Leon-Medina J.X., Anaya M., Pozo F., Tibaduiza D.A. Application of manifold learning algorithms to improve the classification performance of an electronic nose. Proceedings of the 2020 IEEE International Instrumentation and Measurement Technology Conference (I2MTC).

[18] Leon-Medina J.X., Pineda-Muñoz W.A., Tibaduiza Burgos D.A. (2020). Joint Distribution Adaptation for Drift Correction in Electronic Nose Type Sensor Arrays. IEEE Access.

[19] Leon-Medina J.X., Anaya M., Pozo F., Tibaduiza D. (2020). Nonlinear Feature Extraction Through Manifold Learning in an Electronic Tongue Classification Task. Sensors.

[20] Li L., Zhou H., Liu H., Zhang C., Liu J. (2020). A hybrid method coupling empirical mode decomposition and a long short-term memory network to predict missing measured signal data of SHM systems. Struct. Health Monit..

[21] Ayesha S., Hanif M.K., Talib R. (2020). Overview and comparative study of dimensionality reduction techniques for high dimensional data. Inf. Fusion.

[22] Turchetti C., Falaschetti L. (2019). A manifold learning approach to dimensionality reduction for modeling data. Inf. Sci..

[23] Zhang L., Wang X., Huang G.B., Liu T., Tan X. (2018). Taste recognition in e-tongue using local discriminant preservation projection. IEEE Trans. Cybern..

[24] Avci O., Abdeljaber O., Kiranyaz S., Hussein M., Gabbouj M., Inman D.J. (2021). A review of vibration-based damage detection in civil structures: From traditional methods to Machine Learning and Deep Learning applications. Mech. Syst. Signal Process..

[25] Gardner P., Fuentes R., Dervilis N., Mineo C., Pierce S., Cross E., Worden K. (2020). Machine learning at the interface of structural health monitoring and non-destructive evaluation. Philos. Trans. R. Soc. A.

[26] Chandrasekhar K., Stevanovic N., Cross E.J., Dervilis N., Worden K. (2021). Damage detection in operational wind turbine blades using a new approach based on machine learning. Renew. Energy.

[27] Flah M., Nunez I., Chaabene W.B., Nehdi M.L. (2020). Machine learning algorithms in civil structural health monitoring: A systematic review. Arch. Comput. Methods Eng..

[28] Kumar K., Biswas P.K., Dhang N. (2020). Time series-based SHM using PCA with application to ASCE benchmark structure. J. Civ. Struct. Health Monit..

[29] Singh S.K., Sikdar S., Malinowski P.H. (2021). An optimized data fusion strategy for structural damage assessment using electromechanical impedance. Smart Mater. Struct..

[30] Sarmadi H., Karamodin A. (2020). A novel anomaly detection method based on adaptive Mahalanobis-squared distance and one-class kNN rule for structural health monitoring under environmental effects. Mech. Syst. Signal Process..

[31] Outa R., Chavarette F.R., Mishra V.N., Gonçalves A.C., Roefero L.G., Moro T.C. (2020). Prognosis and fail detection in a dynamic rotor using artificial immunological system. Eng. Comput..

[32] Agrawal A.K., Chakraborty G. (2021). On the use of acquisition function-based Bayesian optimization method to efficiently tune SVM hyperparameters for structural damage detection. Struct. Control. Health Monit..

[33] Rahbari A., Rébillat M., Mechbal N., Canu S. (2021). Unsupervised damage clustering in complex aeronautical composite structures monitored by Lamb waves: An inductive approach. Eng. Appl. Artif. Intell..

[34] Agis D., Tibaduiza D.A., Pozo F. (2020). Vibration-based detection and classification of structural changes using principal component analysis and t-distributed stochastic neighbor embedding. Struct. Control. Health Monit..

[35] Zugasti E. (2014). Design and Validation of a Methodology for Wind Energy Structures Health Monitoring. Ph.D. Thesis.

[36] Hoxha E., Vidal Seguí Y., Pozo Montero F. Supervised classification with SCADA data for condition monitoring of wind turbines. Proceedings of the 9th ECCOMAS Thematic Conference on Smart Structures and Materials.

[37] Pozo F., Vidal Y., Salgado Ó. (2018). Wind turbine condition monitoring strategy through multiway PCA and multivariate inference. Energies.

[38] Agis Cherta D., Vidal Seguí Y., Pozo Montero F. Damage diagnosis for offshore fixed wind turbines. Proceedings of the 17th Conference on Renewable Energy and Power Quality.

[39] Anaya M., Tibaduiza D.A., Pozo F. (2015). A bioinspired methodology based on an artificial immune system for damage detection in structural health monitoring. Shock Vib..

[40] Cayton L. (2005). Algorithms for manifold learning. Univ. Calif. San Diego Tech. Rep..

[41] Chen T., Guestrin C. Xgboost: A scalable tree boosting system. Proceedings of the 22nd ACM Sigkdd International Conference On Knowledge Discovery and Data Mining.

[42] Friedman J.H. (2001). Greedy function approximation: A gradient boosting machine. Ann. Stat..

[43] Brownlee J. (2016). XGBoost With Python: Gradient Boosted Trees with XGBoost and Scikit-Learn.

[44] Chang Y.C., Chang K.H., Wu G.J. (2018). Application of eXtreme gradient boosting trees in the construction of credit risk assessment models for financial institutions. Appl. Soft Comput..

[45] Hastie T., Tibshirani R., Friedman J. (2009). The Elements of Statistical Learning: Data Mining, Inference, and Prediction.

[46] Wong T.T. (2015). Performance evaluation of classification algorithms by k-fold and leave-one-out cross validation. Pattern Recognit..

[47] Sokolova M., Lapalme G. (2009). A systematic analysis of performance measures for classification tasks. Inf. Process. Manag..

[48] Encalada-Dávila Á., Puruncajas B., Tutivén C., Vidal Y. (2021). Wind Turbine Main Bearing Fault Prognosis Based Solely on SCADA Data. Sensors.

[49] Mújica L.E., Ruiz M., Pozo F., Rodellar J., Güemes A. (2013). A structural damage detection indicator based on principal component analysis and statistical hypothesis testing. Smart Mater. Struct..

